# Novel *GRHL2* Gene Variant Associated with Hearing Loss: A Case Report and Review of the Literature

**DOI:** 10.3390/genes12040484

**Published:** 2021-03-26

**Authors:** Katarina Trebusak Podkrajsek, Tine Tesovnik, Nina Bozanic Urbancic, Saba Battelino

**Affiliations:** 1 Institute of Biochemistry and Molecular Genetics, Faculty of Medicine, University of Ljubljana, Vrazov trg 2, 1000 Ljubljana, Slovenia; katarina.trebusakpodkrajsek@mf.uni-lj.si; 2 University Medical Centre Ljubljana, University Children’s Hospital, Bohoriceva 20, 1000 Ljubljana, Slovenia; tine.tesovnik@kclj.si; 3 Department of Otorhinolaryngology and Cervicofacial Surgery, University Medical Centre Ljubljana, Zaloska 2, 1000 Ljubljana, Slovenia; nina.bozanic@kclj.si; 4 Faculty of Medicine, University of Ljubljana, Vrazov trg 2, 1000 Ljubljana, Slovenia

**Keywords:** autosomal-dominant hearing loss, NGS, next-generation sequencing, GRHL2

## Abstract

In contrast to the recessive form, hearing loss inherited in a dominant manner is more often post-lingual and typically results in a progressive sensorineural hearing loss with variable severity and late onset. Variants in the *GRHL2* gene are an extremely rare cause of dominantly inherited hearing loss. Genetic testing is a crucial part of the identification of the etiology of hearing loss in individual patients, especially when performed with next-generation sequencing, enabling simultaneous analysis of numerous genes, including those rarely associated with hearing loss. We aimed to evaluate the genetic etiology of hearing loss in a family with moderate late-onset hearing loss using next-generation sequencing and to conduct a review of reported variants in the *GRHL2* gene. We identified a novel disease-causing variant in the *GRHL2* gene (NM_024915: c.1510C>T; p.Arg504Ter) in both affected members of the family. They both presented with moderate late-onset hearing loss with no additional clinical characteristics. Reviewing known *GRHL2* variants associated with hearing loss, we can conclude that they are more likely to be truncating variants, while the associated onset of hearing loss is variable.

## 1. Introduction

Inherited dominant hearing loss accounts for approximately 20% of non-syndromic hearing loss. In contrast to the recessive form, it is more often post-lingual [1] and typically results in a progressive sensorineural hearing loss with variable severity and onset between the ages 10 and 40 years [2]. Genetic testing is a crucial part of the identification of the etiology of hearing loss in individual patients, especially when performed with next-generation sequencing (NGS), enabling simultaneous analysis of numerous genes, including those rarely associated with hearing loss. It can be directed toward the panel of genes associated with non-syndromic or syndromic hearing loss and sequencing of the exome or whole genome [3]. In recent years, the number of genes associated with hearing loss has become extremely broad, and there are currently more than 50 genetic loci associated with the autosomal dominant form of hearing loss [4].

Grainyhead-like 2 (*GRHL2*) gene, also known as transcription factor cellular promoter 2-like 3 (*TFCP2L3*) (OMIM *608576), is an encoding transcription factor expressed in human epithelial tissues [5]. It regulates epithelial morphogenesis and has an important function in the regulation of tight-junction-associated components [5]. It was first associated with the autosomal dominant form of hearing loss (DFNA28) in 2002 in a North American family affected with mild to moderate post-lingual progressive bilateral sensorineural hearing loss [6]. The association was later confirmed in another multi-generation family with post-lingual hearing loss, with a highly variable age of onset and progression [7]. In addition, *GRHL2* variants have been associated with age-related hearing impairment [8]. The gene has also been associated with syndromes unrelated to hearing loss, namely an autosomal-recessive ectodermal dysplasia syndrome in individuals coming from two unrelated consanguineous Kuwaiti families [9] and autosomal-dominant posterior polymorphous corneal dystrophy-4 in Czech and British families [10].

This study aimed to evaluate the genetic etiology of hearing loss in two members of a family with moderate late-onset hearing loss and to conduct a review of the known *GRHL2* variants associated with hearing loss.

## 2. Materials and Methods

The patient and her mother were examined at the outpatient clinic of the Department of Otorhinolaryngology and Cervicofacial Surgery, University Medical Centre Ljubljana. They both gave their written informed consent before the study. The study followed the principles of the Declaration of Helsinki. The evaluation comprised a detailed family history; a medical history focusing on potential causes of acquired hearing loss; and complete audiological history, including the age of onset of hearing loss, the rate of progression, and other audiological and vestibular symptoms.

Genetic analysis was performed at the Clinical Institute for Special Laboratory Diagnostics of the University Children’s Hospital, University Medical Centre Ljubljana. We isolated genomic DNA from peripheral blood samples with an established protocol using FlexiGene DNA Kit 250 (Qiagen, Hilden, Germany). Targeted NGS with a TruSight One Sequencing Panel was performed on the MiSeq platform desktop sequencer using MiSeq Reagent kit v3 (all Illumina, Hayward, CA, USA). The collected NGS data were analyzed using the bcbio-nextgen toolkit, Version 1.2.3 [11]. Bioinformatics data analysis included Burrows-Wheeler and Maximal Exact Match aligner (BWA-MEM) [12] (human genome reference: GRCh37), GATK HaplotypeCaller [13], Freebayes [14], Strelka2 [15], and VarDict [16] variant callers. Ensemble final variant datasets included variants called by at least two variant callers and filtered by variant quality score according to Genome Analysis Toolkit (GATK) best practices. The variant annotation and filtration were performed using Variant Annotation and Filter Tool (VarAFT; Version 2.17) [17]. Rare variants with a minor allele frequency less than 5% in genes reported to be related to syndromic and non-syndromic hearing loss were further evaluated; the gene list was previously reported in [18]. Further variant filtering and selection of candidate variants were done on the basis of data available in The Human Gene Mutation Database Professional (HGMD) [19], the Deafness Variation Database Version 8.2.1 (DVD) (deafnessvariationdatabase.org) [20], and Clinvar [21]. Inheritance and clinical presentation associated with the gene harboring an individual variant were taken into account. Novel variants were evaluated using in silico prediction tools. The pathogenicity of the variants was evaluated as recommended by the American College of Medical Genetics (ACMG) [22]. Possible causative variants were further confirmed in the patient and her mother by Sanger sequencing using custom oligonucleotides, BigDye Terminator v3.1 sequencing kit, and ABI Genetic Analyser 3500 (both Applied Biosystems, Foster City, CA, USA).

## 3. Results

### 3.1. Clinical Presentation

**Figure 1:**
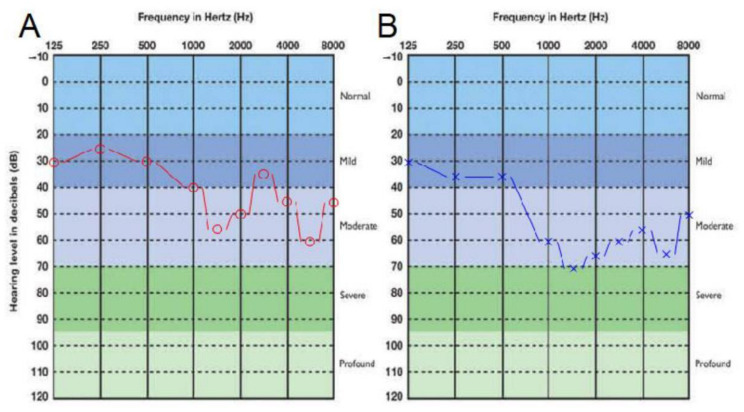
Pure-tone audiogram data of the daughter at first admittance: (**A**) right ear and (**B**) left ear. Red and blue line depict air conduction hearing threshold.

Both patients in the family, mother and daughter, had late-onset hearing loss. The 29-year-old daughter’s first visit to the otorhinolaryngology outpatient clinic was at the age of 28. At the time, she complained of being hard of hearing, which was primarily impeding her social encounters and causing everyday life limitations. She had no other otorhinolaryngological or other health complaints. In her otorhinolaryngological status, otomicroscopy revealed normal eardrums and no other pathological findings, while she had type A tympanometric curves in both ears. Pure-tone audiometry was performed in a standard environment for both ears at 0.25, 0.5, 1, 2, 4, 6, and 8 kHz for air conduction and at 0.5, 1, 2, and 4 kHz for bone conduction. There was no air–bone gap between the curves, and symmetrical sensorineural hearing loss in a W shape between 35–55–35–60 dB was diagnosed (Figure 1). In vestibular testing, caloric testing and a video head impulse test revealed good symmetrical function of the vestibular part of the inner ear. Magnetic resonance imaging showed no pathological changes. Genetic testing with NGS was directed, and two hearing aids were prescribed, and her hearing with amplification improved to 15–30 dB. She had no additional health-related problems. 

**Figure 2:**
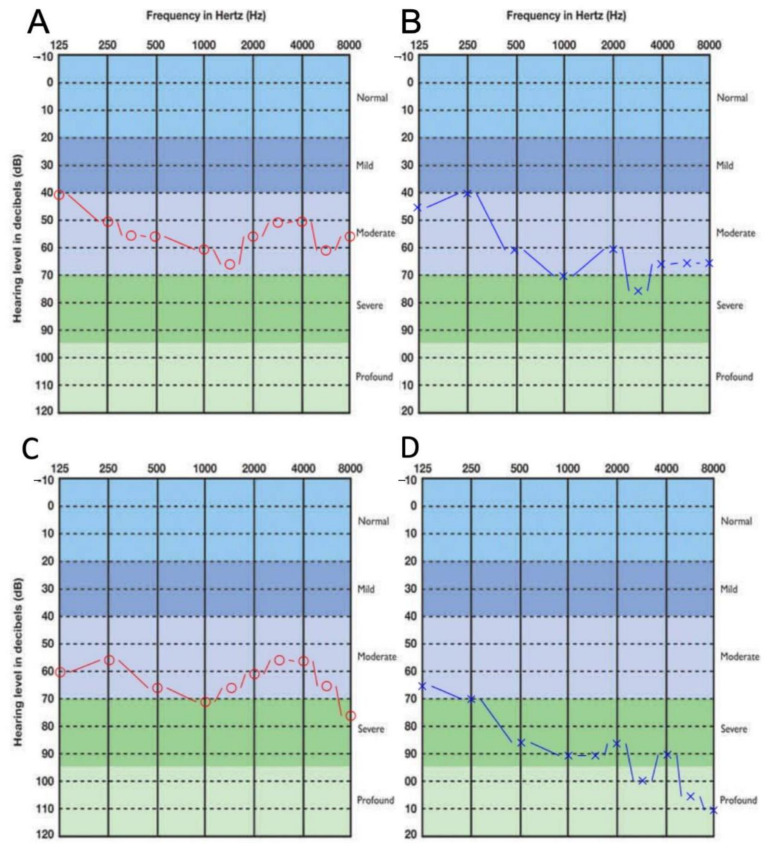
Pure-tone audiogram data of the mother at first admittance (**A,** right ear; **B,** left ear) and after 14 years, at age 53 years (**C**, right ear; **D**, left ear;). Red and blue lines depict air conduction hearing thresholds.

Her mother, age 53, had her first otorhinolaryngology exam when she was 39 years old. At the time, pure-tone audiometry revealed no air–bone gap between the curves, and symmetrical sensorineural hearing loss in a W shape between 30–55–35–60 dB was diagnosed, as had been diagnosed in her daughter (Figure 2). Vestibular testing revealed good symmetrical function of the vestibular part of the inner ear. Magnetic resonance imaging showed no pathological changes. Two hearing aids were prescribed, and hearing with amplification improved to 15–30 dB. She had had only primary school education and was exposed to occupational noise. Her hearing deteriorated in the following 14 years to asymmetrical hearing loss from 75–70–55 dB on the right to 85–100 dB on the left side. She had no additional health-related problems. However, she was recently diagnosed with anaplastic lymphoma kinase (ALK)-positive lung adenocarcinoma. 

### 3.2. Genetic Analysis

**Figure 3:**
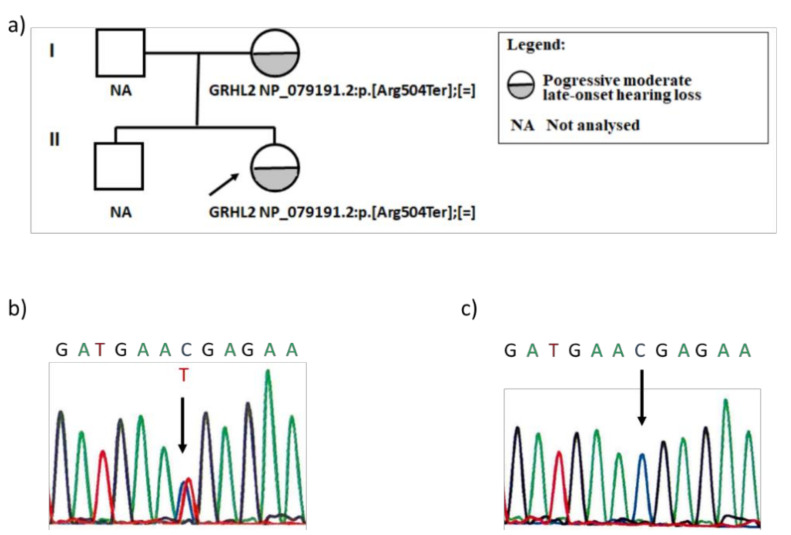
Family pedigree (**a**) and electropherogram of the nucleotide sequence of the *GRHL2* gene exon 12 encompassing novel variant NM_024915: c.1510C>T (NP_079191.2: p.Arg504Ter) (**b**); normal sequence (**c**).

Following variant filtering and selection of the candidate variants, we identified a heterozygous missense variant NM_024915: c.1510C>T in the *GRHL2* gene as a candidate pathogenic variant in the index patient. The same variant was identified in her mother using Sanger sequencing (Figure 3). The genetic variant introduced a premature termination codon at the amino acid position 504 (NP_079191.2: p.Arg504Ter). So far, this variant has not been reported in patients with hearing loss or in the general population, as reported by gnomAd [23]. In silico tools predicted it to be causative (MutationTaster: 1; CADD: score 46). According to ACMG recommendations [22], it was classified as likely pathogenic with the following grades: PVS1, null variant in a gene where the loss of function is a known mechanism of disease, and PM2, extremely low frequency in gnomAD population databases. No additional *GRHL2* variants were identified, while the coverage of the *GRHL2* gene in the NGS sequencing was perfect.

## 4. Discussion

The importance of genetic testing in hearing loss is not deniable. In addition to enabling identification of the genetic etiology and informed genetic counselling, it facilitates clinical interventions and targeted clinical monitoring to identify comorbidities, as reviewed in [18]. The known genetic etiology of hearing loss can also be an important factor when choosing a profession since it can, in some cases, predict the deterioration of hearing related to a noisy working environment [24]. In the family reported here, we identified a novel variant in the *GRHL2* gene predicted to be related to late-onset hearing loss inherited in a dominant manner. 

**Figure 4:**
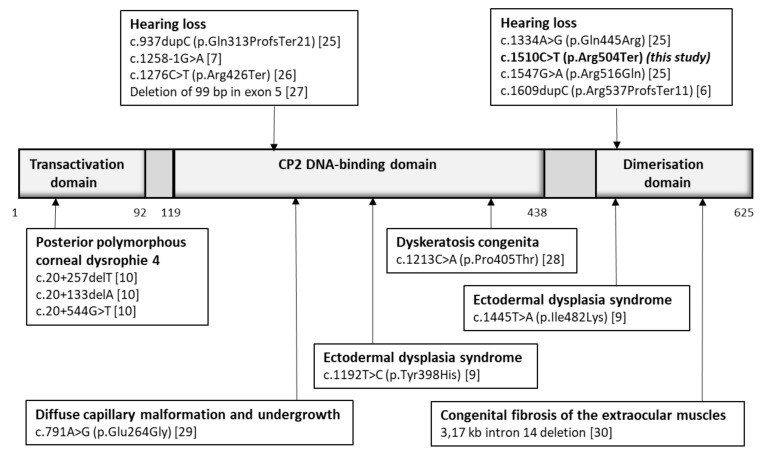
Schematic presentation of the functional domains of the GRHL2 protein, with the location of known disease-causing variants grouped by specific disorder (reference sequences for the variants or amino acid numbering of the protein domains were NM_024915 and NP_079191.2).

The *GRHL2* gene is an extremely rare cause of hearing loss. Currently, according to the professional version of the HGMD [19], there are only seven disease-causing variants reported to be associated with hearing loss inherited in an autosomal dominant manner. The variants and their locations in relation to the GRHL2 protein domains are summarized in Figure 4. The majority of them introduce a premature termination codon, namely c.937dupC (p.Gln313ProfsTer21) [25], c.1276C>T (p.Arg426Ter) [26], or c.1609dupC (p.Arg537ProfsTer11) [6], or result in the deletion of part of exon 5 [27]. There is only one splicing variant c.1258-1G>A [7], located in the cryptic 3′ splice site, and two missense variants c.1334A>G (p.Gln445Arg) and c.1547G>A (p.Arg516Gln) [25] located in the DNA-binding domain or dimerization domain of the protein. GRHL2 is a transcriptional factor [5]**,** so those domains are crucial for its function. The majority of hearing-loss-related variants are truncating variants, while missense and deep intronic variants seem to be more likely to be associated with other *GRHL2*-related pathologies [9,10,28,29,30]. In zebrafish, loss of *GRHL2b* induces midbrain–hindbrain boundary and otic vesicle defects [31]. It is therefore reasonable to assume that any loss-of-function variant might lead to similar consequences. Nevertheless, a study that functionally evaluates a larger number of *GRHL2* gene variants is required.

The clinical presentation of *GRHL2*-related hearing loss is reported to be moderate, progressive, bilateral, and late-onset [6,7,25,26]. In patients with the splice site *GRHL2* variant, the onset was in middle to late adulthood [7], while in patients with the truncating variant c.1609dupC (p.Arg537ProfsTer11), it was variable, with the earliest onset at the age of 7 years [6]. Both patients reported here with the novel truncating variant had had their hearing loss diagnosed in their early adulthood, the daughter when she was 28 and the mother when she was 39, which is consistent with the reported onset in *GRHL2* [7]. Even though not documented, their hearing might have been affected earlier, since they both had had poor school performance and were only able to successfully conclude middle school (the daughter) or primary school (the mother). Both patients reported here had similar audiometric W-shaped curves, but they had no tinnitus, as can be seen in some hearing loss types with genetic etiology [26]. The progressive nature of the hearing loss cannot be confirmed in the daughter since hearing loss was documented only for a little more than a year, but it was documented in her mother (Figure 2), and it is consistent with previous reports [6,7]. Nevertheless, the severity of her hearing loss might be due to the combined genetic *GRHL2* defect and an environmental cause, since she was exposed to occupational noise and some *GRHL2* variants have previously been associated with noise-induced hearing loss in a Chinese population [24].

Other than hearing loss, the patients reported here had no additional clinical characteristics reported to be associated with the *GRHL2* genetic defect, such as diffuse capillary malformations and undergrowth [29], ectodermal dysplasia [9], or eye abnormalities [28]. Recent studies have also demonstrated the role of the GRHL family of proteins in carcinogenesis, as reviewed in [32,33], specifically GRHL2 in human breast cancer [34]. However, at the age of 30, the index patient reported here had no history of breast cancer or other malignant diseases to corroborate this observation. On the other hand, the mother, also a carrier of the *GRHL2* variant, was recently diagnosed with ALK-positive lung adenocarcinoma. Studies have reported that patients with high *GRHL2* expression are associated with a poor prognosis in small-cell lung cancer [35]. However, the influence of reduced expression due to a truncating genetic variant on the disease development was not evaluated.

## 5. Conclusions

We have reported a novel truncating disease-causing variant in the *GRHL2* gene related to moderate late-onset hearing loss with no additional clinical characteristics. Reviewing the known *GRHL2* variants associated with hearing loss, it can be concluded that they are more likely to be truncating variants, while the associated onset of hearing loss is variable.

## Data Availability

The data presented in this study are available on request from the corresponding author. The data are not publicly available according to the National Medical Ethics Committee Consent (Nr. 21/06/15).

## References

[1] Chang K.W. (2015). Genetics of Hearing Loss-Nonsyndromic. Otolaryngol. Clin. N. Am..

[2] Liu X., Xu L., Zhang S., Xu Y. (1994). Epidemiological and Genetic Studies of Congenital Profound Deafness in the General Population of Sichuan, China. Am. J. Med. Genet..

[3] Parker M., Bitner-Glindzicz M. (2015). Genetic investigations in childhood deafness. Arch. Dis. Child..

[4] Van Camp G., Smith R.J.H. Hereditary Hearing Loss Homepage. https://hereditaryhearingloss.org.

[5] Werth M., Walentin K., Aue A., Schönheit J., Wuebken A., Pode-Shakked N., Vilianovitch L., Erdmann B., Dekel B., Bader M. (2010). The transcription factor grainyhead-like 2 regulates the molecular composition of the epithelial apical junctional complex. Development.

[6] Peters L.M., Anderson D.W., Griffith A.J., Grundfast K.M., San Agustin T.B., Madeo A.C., Friedman T.B., Morell R.J. (2002). Mutation of a transcription factor, TFCP2L3, causes progressive autosomal dominant hearing loss, DFNA28. Hum. Mol. Genet..

[7] Vona B., Nanda I., Neuner C., Müller T., Haaf T. (2013). Confirmation of GRHL2 as the gene for the DFNA28 locus. Am. J. Med. Genet. Part A.

[8] Van Laer L., Van Eyken E., Fransen E., Huyghe J.R., Topsakal V., Hendrickx J.J., Hannula S., Mäki-Torkko E., Jensen M., Demeester K. (2008). The grainyhead like 2 gene (GRHL2), alias TFCP2L3, is associated with age-related hearing impairment. Hum. Mol. Genet..

[9] Petrof G., Nanda A., Howden J., Takeichi T., McMillan J.R., Aristodemou S., Ozoemena L., Liu L., South A.P., Pourreyron C. (2014). Mutations in GRHL2 result in an autosomal-recessive ectodermal dysplasia syndrome. Am. J. Hum. Genet..

[10] Liskova P., Dudakova L., Evans C.J., Rojas Lopez K.E., Pontikos N., Athanasiou D., Jama H., Sach J., Skalicka P., Stranecky V. (2018). Ectopic GRHL2 Expression Due to Non-coding Mutations Promotes Cell State Transition and Causes Posterior Polymorphous Corneal Dystrophy 4. Am. J. Hum. Genet..

[11] Bcbio-Nextgen. https://github.com/bcbio/bcbio-nextgen.

[12] Li H. (2013). Aligning sequence reads, clone sequences and assembly contigs with BWA-MEM. arXiv.

[13] McKenna A., Hanna M., Banks E., Sivachenko A., Cibulskis K., Kernytsky A., Garimella K., Altshuler D., Gabriel S., Daly M. (2010). The genome analysis toolkit: A MapReduce framework for analyzing next-generation DNA sequencing data. Genome Res..

[14] Garrison E., Marth G. (2012). Haplotype-based variant detection from short-read sequencing—Free bayes—Variant Calling—Longranger. arXiv.

[15] Kim S., Scheffler K., Halpern A.L., Bekritsky M.A., Noh E., Källberg M., Chen X., Kim Y., Beyter D., Krusche P. (2018). Strelka2: Fast and accurate calling of germline and somatic variants. Nat. Methods.

[16] Lai Z., Markovets A., Ahdesmaki M., Chapman B., Hofmann O., Mcewen R., Johnson J., Dougherty B., Barrett J.C., Dry J.R. (2016). VarDict: A novel and versatile variant caller for next-generation sequencing in cancer research. Nucleic Acids Res..

[17] Desvignes J.P., Bartoli M., Delague V., Krahn M., Miltgen M., Béroud C., Salgado D. (2018). VarAFT: A variant annotation and filtration system for human next generation sequencing data. Nucleic Acids Res..

[18] Urbančič N.B., Battelino S., Tesovnik T., Podkrajšek K.T. (2020). The importance of early genetic diagnostics of hearing loss in children. Medicina.

[19] Human Gene Mutation Database. http://www.hgmd.org/.

[20] Deafness Variation Database. http://deafnessvariationdatabase.org/.

[21] Landrum M.J., Chitipiralla S., Brown G.R., Chen C., Gu B., Hart J., Hoffman D., Jang W., Kaur K., Liu C. (2020). ClinVar: Improvements to accessing data. Nucleic Acids Res..

[22] Richards S., Aziz N., Bale S., Bick D., Das S., Gastier-Foster J., Grody W.W., Hegde M., Lyon E., Spector E. (2015). Standards and guidelines for the interpretation of sequence variants: A joint consensus recommendation of the American College of Medical Genetics and Genomics and the Association for Molecular Pathology. Genet. Med..

[23] The Genome Aggregation Database. http://gnomad.broadinstitute.org//.

[24] Zhang X., Liu Y., Zhang L., Yang Z., Yang L., Wang X., Jiang C.X., Wang Q., Xia Y., Chen Y. (2015). Associations of genetic variations in EYA4, GRHL2 and DFNA5 with noise-induced hearing loss in Chinese population: A case-control study. Environ. Health.

[25] Iwasa Y.I., Nishio S.Y., Usami S.I. (2016). Comprehensive genetic analysis of Japanese autosomal dominant sensorineural hearing loss patients. PLoS ONE.

[26] Wu D., Huang W., Xu Z., Li S., Zhang J., Chen X., Tang Y., Qiu J., Wang Z., Duan X. (2020). Clinical and genetic study of 12 Chinese Han families with nonsyndromic deafness. Mol. Genet. Genom. Med..

[27] Ji H., Lu J., Wang J., Li H., Lin X. (2014). Combined examination of sequence and copy number variations in human deafness genes improves diagnosis for cases of genetic deafness. BMC Ear Nose Throat Disord..

[28] Walne A.J., Collopy L., Cardoso S., Ellison A., Plagnol V., Albayrak C., Albayrak D., Kilic S.S., Patıroglu T., Akar H. (2016). Marked overlap of four genetic syndromes with dyskeratosis congenita confounds clinical diagnosis. Haematologica.

[29] Cubiró X., Rozas-Muñoz E., Castel P., Roé Crespo E., Garcia-Melendo C., Puig L., Baselga E. (2020). Clinical and genetic evaluation of six children with diffuse capillary malformation and undergrowth. Pediatr. Dermatol..

[30] Abu-Amero K.K., Kondkar A.A., Khan A.O. (2017). A microdeletion in the GRHL2 Gene in two unrelated patients with congenital fibrosis of the extra ocular muscles. BMC Res. Notes.

[31] Dworkin S., Darido C., Georgy S.R., Wilanowski T., Srivastava S., Ellett F., Pase L., Han Y., Meng A., Heath J.K. (2012). Midbrain-hindbrain boundary patterning and morphogenesis are regulated by diverse grainy head-like 2-dependent pathways. Development.

[32] Sundararajan V., Pang Q.Y., Choolani M., Huang R.Y.J. (2020). Spotlight on the Granules (Grainyhead-Like Proteins)—From an Evolutionary Conserved Controller of Epithelial Trait to Pioneering the Chromatin Landscape. Front. Mol. Biosci..

[33] He J., Feng C., Zhu H., Wu S., Jin P., Xu T. (2020). Grainyhead-like 2 as a double-edged sword in development and cancer. Am. J. Transl. Res..

[34] Leth-Larsen R., Terp M.G., Christensen A.G., Elias D., Kühlwein T., Jensen O.N., Petersen O.W., Ditzel H.J. (2012). Functional heterogeneity within the CD44 high human breast cancer stem cell-like compartment reveals a gene signature predictive of distant metastasis. Mol. Med..

[35] Pan X., Zhang R., Xie C., Gan M., Yao S., Yao Y., Jin J., Han T., Huang Y., Gong Y. (2017). GRHL2 suppresses tumor metastasis via regulation of transcriptional activity of rhog in non-small cell lung cancer. Am. J. Transl. Res..

